# Application of Machine Learning in Microbiology

**DOI:** 10.3389/fmicb.2019.00827

**Published:** 2019-04-18

**Authors:** Kaiyang Qu, Fei Guo, Xiangrong Liu, Yuan Lin, Quan Zou

**Affiliations:** ^1^College of Intelligence and Computing, Tianjin University, Tianjin, China; ^2^School of Information Science and Technology, Xiamen University, Xiamen, China; ^3^Department of System Integration, Sparebanken Vest, Bergen, Norway; ^4^Institute of Fundamental and Frontier Sciences, University of Electronic Science and Technology of China, Chengdu, China; ^5^Center for Informational Biology, University of Electronic Science and Technology of China, Chengdu, China

**Keywords:** microorganisms, classification, environment, species, association, diseases

## Abstract

Microorganisms are ubiquitous and closely related to people’s daily lives. Since they were first discovered in the 19th century, researchers have shown great interest in microorganisms. People studied microorganisms through cultivation, but this method is expensive and time consuming. However, the cultivation method cannot keep a pace with the development of high-throughput sequencing technology. To deal with this problem, machine learning (ML) methods have been widely applied to the field of microbiology. Literature reviews have shown that ML can be used in many aspects of microbiology research, especially classification problems, and for exploring the interaction between microorganisms and the surrounding environment. In this study, we summarize the application of ML in microbiology.

## Introduction

Microorganisms first appeared approximately 3.5 billion years ago, making them one of the earliest living things on Earth (Nannipieri et al., 2010). Microorganisms include bacteria, viruses, fungi, some small protozoa, and microscopic algae. These organisms, which are closely related to human beings (Ley et al., 2006a), have a wide range of beneficial and harmful uses, including in the food (Cotter et al., 2005), medicine (Petrof et al., 2012; Yu et al., 2018), agriculture (Morris et al., 1986), industrial (Souza, 2010), environmental protection and other fields (Reiff and Kelly, 2010).

Microbiology is a discipline that studies the structure and function of microbial groups, the interrelationships and mechanisms of internal communities, and the relationships between microorganisms and their environments or hosts (Alexander, 1962; Niel, 1966). The microbiome is a collection of all microbial species and their genetic information and functions in a given environment. Studies of the microbiome also include the interaction between different microorganisms (DiMucci et al., 2018), the interaction between microorganisms and other species (Xie et al., 2018), and the interaction between microorganisms and the environment (Moitinho-Silva et al., 2017). Because of their small size, the microscope is an important tool for studying microorganisms. However, microscopy analyses only allow observation and must therefore be complemented by culture techniques to study the biological, physiological, genetic, metabolic, pathogenic and other biological characteristics of microorganisms (Waldron, 2018). During cultivation, researchers can also explore the interactions between microorganisms and the environment, which reflect the breadth and diversity of microbial distribution. A variety of microorganisms living in different environments or in different hosts form microbial communities, which have extensive and complex interactions with the environment and the host and form various types of ecosystems (Srinivasan et al., 2012; Xie et al., 2018).

**Figure 1:**
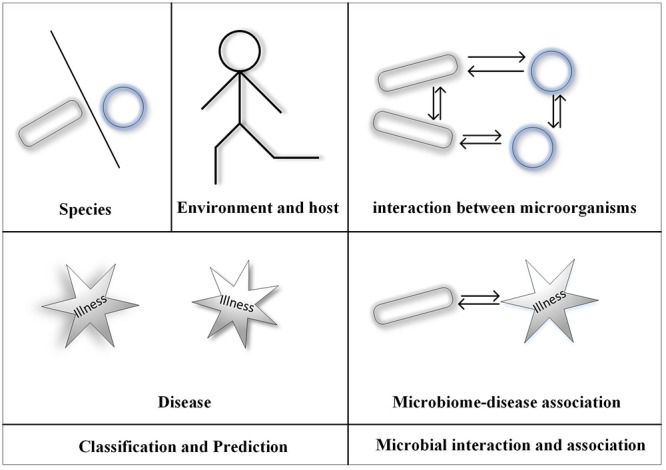
The framework of this paper.

With the development of microbial sequencing in recent years, the microbiome has become increasingly popular in many studies. High-throughput sequencing technology has resulted in generation of an increasing amount of microbial data. Traditional methods using microscopes and biological cultures are expensive and labor intensive; therefore, machine-learning methods have been gradually applied to microbial studies (Huang Y. A. et al., 2017; Huang Z. A. et al., 2017; Wang et al., 2017; Wei et al., 2017a,b; Peng et al., 2018; Yang et al., 2018b; Zou et al., 2018a). Here, we introduce the application of machine learning (ML) in microbial analyses. Since ML is mainly applied to classification and interaction problems, we focus on these two areas. Figure 1 shows the framework of this paper.

## Machine Learning Methods

**Figure 2:**
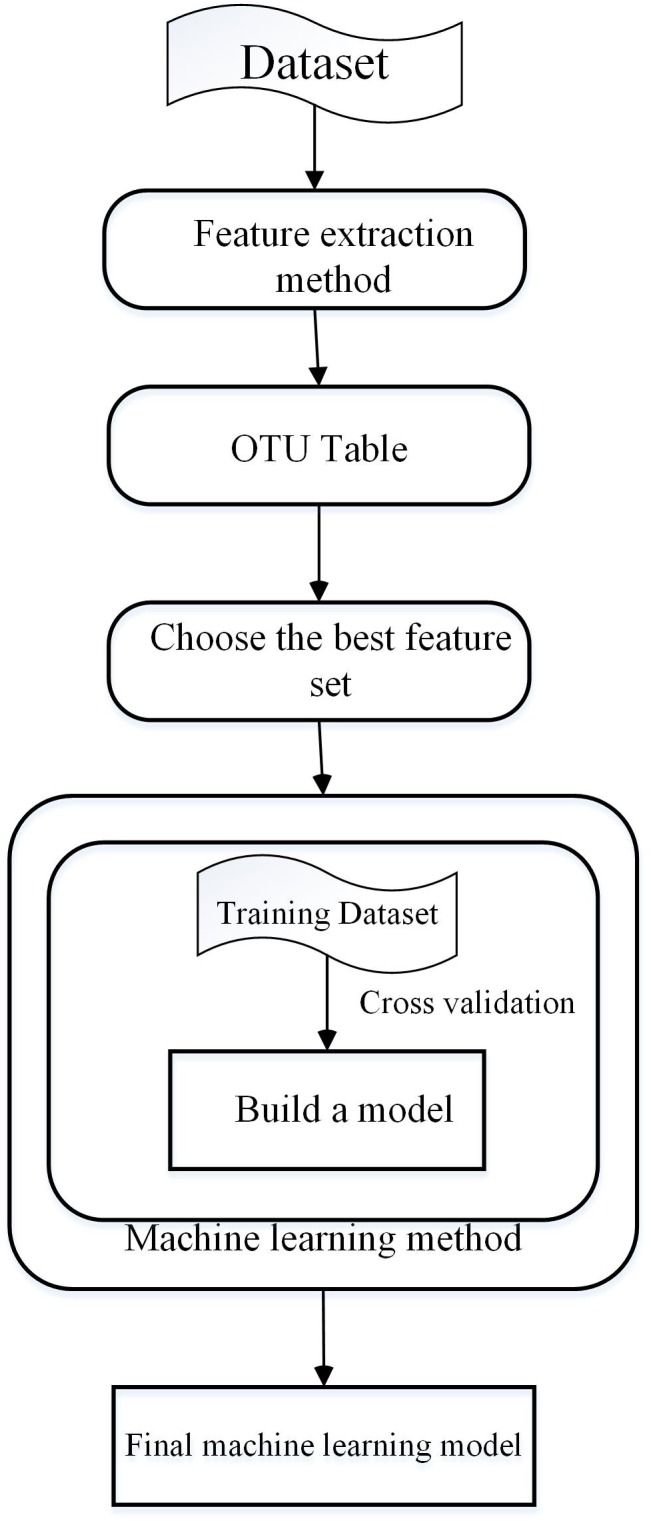
The main steps of machine learning in microbiology.

Machine Learning is a multi-disciplinary subject involving many disciplines including probability theory, statistics, approximation theory, convex analysis, and algorithm complexity theory (Qu et al., 2017; Zou et al., 2018b). ML methods can be divided into two types (Zitnik et al., 2019), supervised learning and unsupervised learning. Supervised learning (Stoter et al., 2019) requires that the model be trained using a training set. The training sets for supervised learning include features and results. Common supervised learning algorithms include regression analysis and statistical classification. Unsupervised learning, also known as clustering, adopts k-means to establish a centriole and reduce error through iteration and descent to achieve classification. With the development of ML, more and more fields have begun to use this technique for research (Chen W. et al., 2016, Chen et al., 2017a,d, 2018a,b,e,f,g; Li et al., 2016; Zou et al., 2016, 2017; Ding et al., 2017a,b; Feng et al., 2017a; Yu et al., 2017a; Zeng et al., 2017a, 2018; Liu et al., 2018; Pan et al., 2018; Wei et al., 2018a,b; Yang et al., 2018a; Zhao et al., 2018b; He et al., 2019; Zhang et al., 2019), for example, drug repositioning (Yu et al., 2016b, 2017b), disease-related microRNA (Chen and Huang, 2017; Chen et al., 2017d, 2018b,e,g; Zhao et al., 2018a,c) identification, and disease-related long non-coding RNA identification (Chen and Yan, 2013; Chen et al., 2017e, 2018c; Hu et al., 2017, 2018). There are four main steps in developing ML algorithms (Oudah and Henschel, 2018). The first step is extraction of the features, which is critical to the ML method (Liu et al., 2015). Then, the operational classification units (OTU) table can be obtained by clustering. Next, important features that can improve the accuracy and efficiency are selected. Finally, a training dataset is used to train the model, after which a test set is used to evaluate the model. The process is summarized in Figure 2.

In microbial studies, according to the collected samples, obtaining relevant OTU is an important step in the study of microbial data. OTU is a type of similar microorganisms, which are cluster according to the similarity DNA sequences (Blaxter et al., 2005). In recent years, OTUs are always used for microbial diversity, especially when analyzing small subunit 16S or 18S rRNA datasets (Schmidt et al., 2014). Sequences can be clustered according to their similarity to one another, and the researcher sets the similarity threshold. After OTU clustering and species classification annotation for OTU, the OTU table can be obtained, which contains the OTU types and quantities for each sample, as well as species annotation information for each OTU.

As we know, some microbes have higher data dimensions, so feature dimensionality reduction is also an important part of data processing. There are some common methods for reducing the dimensionality and many studies are about how to reduce the dimensionality. For example, the principal components analysis (PCA) is a common reduction dimensionality method, which is mainly to decompose the covariance matrix to obtain the principal components and their weights (Jolliffe, 2002). PCA is often used to reduce the dimensionality of dataset while maintaining the feature that maximizes the contribution of the variance in the data set. Principal co-ordinates analysis (PCoA) is another common method. After sorting the feature values and the feature vectors, PCoA selects the features, which are in the top digits and the most significant coordinates in the distance matrix can be found (Podani and Miklós, 2002). The result is a rotation of the data matrix. It does not change the mutual positional relationship between the sample points, but only changes the coordinate system.

In microbial studies, supervised learning is always used, especially the support vector machine (SVM) (Feng et al., 2013a, 2017b; Chen X. X. et al., 2016; Yang et al., 2016), and the Naïve Bayes (NB) (Feng et al., 2013b,c), random forest (RF) (Chen et al., 2018d), and *k* nearest neighbor (KNN) methods (Chen et al., 2017c).

The SVM is a generalized linear classifier that can perform binary classification of data employing a decision basis, according to the maximum-margin hyperplane of the learning sample. The SVM can classify non-linear data by the kernel methods (Drucker et al., 2002). SVM is widely used in bioinformatics, such as the prediction of proteins (Xu et al., 2018a,b,c). The NB method (Meena and Chandran, 2009), which is a classification based on Bayes’ theory and the independent assumption of features that originate from classical mathematical theory (Rodríguez and Kuncheva, 2007), has a solid mathematical foundation and stable classification efficiency. The NB classifier, which requires only a few parameters, is less sensitive to missing data and simpler than other methods (Jordan, 2008). The RF is a classifier that contains multiple decision trees and its output accords to the voting on each decision tree (Svetnik et al., 2003). KNN (Cui et al., 2001) is a theoretically mature method. The method infers the sample category based on its neighbors. The main steps of the algorithm are as follows (Liao and Vemuri, 2002). First, the distance, which is between the test sample and each training sample, should be calculated. Then, the nearest *k* training samples are found as the nearest neighbors of the test sample. Finally, the test sample is classified according to the categories of the *k* nearest neighbors.

## Classification and Prediction in Microbiology

### Prediction of Microbial Species

There are two main types of microorganisms (Maiden et al., 1998), one of them with non-cellular morphology (Yeom and Javidi, 2006), such as viruses, and the other with cellular morphology that can divided into two types, one of them namely prokaryotes (Weinbauer, 2010), such as archaea and eubacteria, and the other namely eukaryotes (Nowrousian, 2010), such as fungi and unicellular algae. Different microorganisms have different characteristics, so it is important to identify the microorganisms properly. There are two main approaches to the identification of microorganisms. In one, the species of an unknown microorganism is determined with the goal of classifying it based on its domain, kingdom, phylum, class, order, family genus and species. In the other, the goal is to determine whether an unknown microorganism belongs to a specific species or not. For example, we can determine if an unknown microorganism is a virus or not, or more specifically, whether it is a certain virus. In this section, we will introduce recent studies that have used machine-learning methods to predict microorganisms.

In the study (Murali et al., 2018), the authors classified specific species of microorganisms using the IDTAXA, which employed the LearnTaxa and IdTaxa functions. Both of these functions are part of the R package DECIPHER, which was released under the GPLv3 license as part of the Bioconductor, which provides tools for the analysis and comprehension of high-throughput genomic data. The LearnTaxa function attempts to reclassify each training sequence into its tagged taxon using a method known as tree descent, which is similar to the decision tree, a commonly ML algorithms. IdTaxa uses the objects returned by the LearnTaxa and query sequences as input data. This system returns the classification results for each sequence in the taxonomic form and provides the relevant confidence for each level. If the confidence does not reaches the required value, which indicates that the classification cannot be accurately performed at that level. The classification of IdTaxa may lead to different conclusions in microbiological studies. Although the misclassification is small, many of the remaining misclassifications may be caused by the errors in the reference taxonomy. Fiannaca et al. (2018) presented a method for identifying the 16S short-read sequences based on *k*-mer and deep learning. According to their results, the method can classify both 16S shotgun (SG) and amplicon (AMP) data very well.

It is important to identify specific microbial sequences in mixed metagenomics samples. At present, gene-based similarity methods are popularly used to classify prokaryotic and host organisms from mixed samples; however, these techniques have major weakness. Therefore, many studies have been conducted to identify better methods for identification of specific microorganisms. Amgarten et al. (2018) proposed a tool known as MARVEL for predicting double-stranded DNA bacteriophage sequences in metagenomics. MARVEL uses the RF method, with a training dataset composed of 1,247 phage and 1,029 bacterial genomes and a test dataset composed of 335 bacteria and 177 phage genomes. The authors proposed six features to identify the phages, then used random forests to select features and found three features provided more information (Grazziotin et al., 2017). Ren et al. (2017) developed VirFinder, which is a ML method based on *k*-mer for virus overlap group identification that avoids gene-based similarity searches. VirFinder trains the ML model through known viral and non-viral (prokaryotic host) sequences to detect the specificity of viral *k*-mer frequencies. The model was trained with host and viral genomes prior to January 1, 2014, and the test set consisted of sequences obtained after January 1, 2014. VirSorter (Roux et al., 2015) is based on reference dependence and reference independence in different kinds of microbial sequence data to identify the viral signal. Experimental results have shown that VirSorter has good performance, especially for predicting viral sequences outside the host genome.

The above methods specifically classify microorganisms according to different needs. When we want to know the taxonomy information of microorganisms, we can use the method, which proposed by Murali et al. (2018). Moreover, MARVEL, VirSort, and VirFinder can identify specific types of microorganisms. According to the Amgarten et al. (2018), these three methods have comparable performance on specificity, but MARVEL has a better recall (sensitivity) performance. We have compiled materials for implementation of the above methods, which are shown in Table 1.

**Table 1:** The available data and materials for prediction of microbial species.

Studies	Availability of data and materials	Reference
IDTAXA	http://DECIPHER.codes	Murali et al., 2018
Fiannaca et al.	https://github.com/IcarPA-TBlab/MetagenomicDC	Fiannaca et al., 2018
MARVEL	https://github.com/LaboratorioBioinformatica/MARVEL	Amgarten et al., 2018
VirFinder	https://github.com/jessieren/VirFinder	Ren et al., 2017
VirSorter	https://github.com/simroux/VirSorter	Roux et al., 2015

### Prediction of Environmental and Host Phenotypes

With the development of next-generation DNA and high-throughput sequencing, a new area of microbiology has been generated. The main research in this field is to link microbial populations to phenotypes and ecological environments, which can provide favorable support for disease outbreaks and precision medicine (Atlas and Bartha, 1981). It is well known that some microorganisms are parasitic and that the surrounding environment and host cells have an important impact on the microbial population. Differences in nutrient availability and environmental conditions lead to differences in microbial communities (Moran, 2015). Because microorganisms can exchange information with the surrounding environment and host cells, we can predict the environmental and host phenotypes based on the microorganisms that are present (Xie et al., 2018). This provides a more comprehensive understanding of the environment and the host, so that we can better use the environment and protect the host. Many studies have recently been conducted to predict environmental and host phenotypes using microorganisms. In this section, we introduce these studies.

Asgari et al. (2018) used shallow subsample representation based on *k*-mer and deep learning, random forests, and SVMs to predict environmental and host phenotypes from 16S rRNA gene sequencing using the MicroPheno system. They found that the shallow subsample representation based on *k*-mer is superior to OTU in terms of body location recognition and Crohn’s disease prediction. In addition, the deep learning method is better than the RF and SVM for large datasets. This method not only can improve the performance, but also avoid overfitting. Moreover, it can reduce the time of pretreatment. Statnikov et al. (2013) used OTUs as an input feature and processed the data as follows. First, the authors sequenced the original DNA, after which they removed the human DNA sequence and defined the OTUs based on the microbial sequence. Next, they quantified the relative abundance of all sequences belonging to each OTU. The authors used SVM, kernel ridge regression, regularized logistic regression, Bayesian logistic regression, the KNN method, the RF method and probabilistic neural networks with different parameters and kernel functions. Overall, they investigated 18 ML methods. In addition, they used five feature extraction methods. The experimental results revealed that the RF, SVM, kernel-regression and Bayesian logic use Laplacian prior regression provided better performance. Based on their research, human skin microorganisms collected from objects that have been touched can be used to identify the individual from which they originated. In this work, the author used a variety of classification and dimensionality reduction methods to explore the effects of each method. It is very useful for the next work, which provides a comprehensive comparison. Schmedes et al. (2018) used the microbial community for forensic identification. In their study, they developed the hidSkinPlex, a novel targeted sequencing method using skin microbiome markers developed for human identification. In forensic science, it is important to estimate the time of death. Johnson et al. (2016) used KNN regression to predict the time interval after death using datasets from nose and ear samples. This indicates that skin microbiota can be an important tool in forensic death investigation. Traditionally, marine biological monitoring involves the classification and morphological identification of large benthic invertebrates, which requires a great deal of time and money. Cordier et al. (2017) used eDNA metabarcoding and supervised ML to build a powerful prediction model of benthic monitoring. Moitinho-Silva et al. (2017), studied the microbial flora of sponges and their HMA-LMA status demonstrated the applicability of ML to exploring host-related microbial community patterns.

Due to the specificity of microbial communities, we can better identify the environment and the host. Moreover, we can judge the existing environmental conditions and host survival status according to the existence of microbial community. We summarize the available datasets and methods, which are shown in Table 2.

**Table 2:** The available data and materials for prediction of environmental and host phenotypes.

Studies	Availability of data and materials	Reference
Asgari et al.	https://llp.berkeley.edu/micropheno	Asgari et al., 2018
Statnikov et al.	https://link.springer.com/article/10.1186/2049-2618-1-11	Statnikov et al., 2013

### Using Microbial Communities to Predict Disease

Microbiomes are important to human health and disease (Bourne et al., 2009). Indeed, there are many microbial communities in the human body. Once a microbial community is out of balance or foreign microorganisms invade, the human body is likely to get sick. For example, intestinal microbial communities are associated with obesity (Ley et al., 2006b) and pulmonary communities with pulmonary infection (Sibley et al., 2008). Because of the complexity of these communities, it is difficult to determine which kind of microbiome communities cause of the disease. Recently, many studies have investigated use of microbiome communities to predict diseases, especially bacterial vaginosis (Srinivasan et al., 2012; Deng et al., 2018) and inflammatory bowel disease (Gillevet et al., 2010). By analyzing microbial communities, we can better understand the disease and then make effective decisions regarding treatment. Therefore, in this section, we discuss current studies investigating use of microbiome communities to predict diseases.

Bacterial vaginosis (BV) is a disease associated with the vaginal microbiome. Beck and Foster (2014) used the genetic algorithm (GP), RF, and logistic regression (LR) to classify BV according to microbial communities. There are two criteria for BV, the Amsel standard, which accord to the discharge, whiff, clue cells, and pH (Amsel et al., 1983), and Nugent score, which dependents on counting gram-positive cells (Nugent et al., 1991). The dataset in Beck et al. study was from Ravel et al. (2011) and Sujatha et al. (2012). The method in the paper (Beck and Foster, 2014) first classifies BV according to vaginal microbiota and related environmental factors, then identifies the most important microbial community for predicting BV.

Hierarchical feature extraction is based on the classification of microbes from kingdoms to species. The existing stratification feature selection algorithm will lead to information loss, and the stratification information of some 16S rRNA sequences is usually incomplete, influencing the classification. Therefore, Oudah and Henschel (2018) proposed a method known as hierarchical feature engineering (HFE) to identify colorectal cancer (CRC). To accomplish this, they used RF, decision trees and the NB method to classify a dataset of Next Generation Sequencing based 16S rRNA sequences provided by metagenomics studies. This method is good for processing datasets with high dimensional features. Moreover, the available dataset and method are in https://github.com/HenschelLab/HierarchicalFeatureEngineering.

In another study (Wisittipanit, 2012), the author focused on predicting inflammatory bowel disease. In that study, patients with Crohn’s disease and ulcerative colitis were compared with healthy controls to identify differences between the mucosa and lumen in different intestinal locations. The author used the Relief algorithm (Kira and Rendell, 1992) to select features, and Metastats (White et al., 2009) to detect differential features. Finally, the author used KNN and SVM as classifiers to perform disease specificity and site specificity analysis.

In this section, we discuss using microorganisms to predict different diseases. Beck and Foster (2014) predicted BV according to the microorganisms and the diagnosis standard of BV. HFE identified the CRC according to the OTU ID and the taxonomy information. Wisittipanit proposed a method to predict Crohn’s disease, based on OTU and feature selection method. The above methods used different ideas to predict diseases by using microorganisms and obtained good results. This indicates that some diseases affect human colonies. According to these colony changes, we can not only predict the disease, but also treat the disease according to the colony condition, which is a direction for future research.

## Interaction and Association in Microbiology

### Interaction Between Microorganisms

The collective behavior of microbial ecosystems in biomes is the result of many interactions between community members. These interactions include metabolite exchange, signaling and quorum sensing processes, as well as growth inhibition and killing (Langille et al., 2013; DiMucci et al., 2018). Understanding the interspecific interactions within microbial communities is critical to understanding the functions of natural ecosystems and the design of synthetic consortia (Mainali et al., 2017). Therefore, in this section, we introduce the application of ML to investigation of interactions between microorganisms.

DiMucci et al. (2018) showed how the microbial interaction network can be combined with the characteristic level of individual microbes to provide an accurate inference of the missing edges in the network and a constructive mechanism of the interaction. The same authors proposed the notion of a composite vector that combined the generated trait vectors and pairwise interactions. The training set for the model is all observed interactions. The model was then used to predict the unobserved interactions. If the random forest classifier is used, feature contributions can be calculated. Microbial interactions in the soil can affect crop yields; therefore, Chang et al. (2017) used the random forest method to predict the productivity based on the microorganisms. In this study, the improved crop productivity differences were linked to the soil microbial composition.

There are cooperative and competitive relationships within the same microbial population. Moreover, there are eight relationships between the different microbial populations, which are neutralism, commensalism, synergism, mutualism, competition, amensalism, parasitism and predation. Understanding the interactions between microorganisms is important for the study of microbial species and for microbial applications. However, there are not many studies on ML in this area, which will be an important research direction.

### Microbiome-Disease Association

There are many kinds of microorganisms in human bodies, and they are inseparable from human health. For example, intestinal microbial disorders can cause intestinal inflammatory diseases (Chen et al., 2017b), such as ulcerative colitis, CRC, atherosclerosis, diabetes and obesity. Accordingly, it is necessary to predict the microbial-disease association because this study not only improves the diagnosis and prognosis of human diseases, but also develops the new drugs (Yu et al., 2015, 2016a; Shi et al., 2016; Su et al., 2018; Fan et al., 2019). However, few studies have investigated predictive analysis of the microbial-disease association. Therefore, in this section, we introduce the application of ML to the study of microbial-disease association.

Fan et al. (2019) proposed a new approach to analyze the microbial-disease association by integrating multiple data sources from the human microbe-disease consortium (MDPH_HMDA) and path-based HeteSim scores. First, heterogeneity networks were constructed. Microbe-disease pair weighting was conducted according to the standardized HeteSim measurement method, after which the microbe-disease-disease pathway and microbe-microbe-disease pathway HeteSim scores were integrated. Finally, the correlation scores of potential micro genome associations were calculated. Xuezhong et al. (2014) proposed a method based on the Human Disease Network (HSDN) in which co-occurrence of disease/symptom terms based on PubMed bibliographic records was used to calculate disease similarity. KATZ (Katz, 1953) is a network based measurement method that calculates the similarly of nodes in a heterogeneous network, to solve the link prediction problem proposed by Katz. The KATZ method has been applied in many fields, including disease-gene association prediction (Xiaofei et al., 2014) and IncRNA-disease association prediction (Chen et al., 2015). Chen et al. (2017b) proposed a novel method based on KATZ to predict associations of human microbiota with non-infectious diseases (named KATZHMDA). The KATZHMDA first constructs adjacency matrix A based on known microbial-disease associations. The kernel similarity matrix KD and KM are calculated based on the disease Gaussian interaction profile and microbial Gaussian interaction profile, respectively. We can construct the integrated matrix A^∗^ based on KM, KD and known microbial-disease associations. Next, all walks of different lengths are integrated to obtain a single microbe-disease association measurement. Therefore, we can calculate microbe-disease association probability in a matrix form. Shi et al. (2018) proposed a prediction method based on binary matrix completion named BMCMDA. The BMCMDA assumes that the incomplete microbiome-disease association (MDA) matrix is the sum of a potential parameterization matrix and a noise matrix. Additionally, the BMCMDA assumes that the independent subscripts of the items observed in the MDA matrix follow the binomial model. Shi et al. (2018) used the same dataset, which was collected from the Human Microbe-Disease Association Database (HMDAD) and included 292 microbes and 39 human diseases, to perform comparisons. According to the study, BMCMDA is better than the KATZHMDA in AUC. BMCMDA can be integrated with other and independent microbial/disease similarities or characteristics to enhance MDA prediction. Moreover, this method can be applied to more prediction aspects. We summarize the available datasets and methods, which are shown in Table 3.

**Table 3:** The available data and materials for microbiome-disease association.

Studies	Availability of data and materials	Reference
Zhou et al.	https://www.nature.com/articles/ncomms5212#supplementary-information	Xiaofei et al., 2014
KATZHMDA	http://dwz.cn/4oX5mS.	Chen et al., 2017b
BMCMDA	https://github.com/JustinShi2016/ISBRA2017	Shi et al., 2018

## Conclusion

Microorganisms are involved in many life activities, and affect their surrounding environment and other organisms. Microorganisms play important roles in human heath, crop growth, livestock farming, environmental management, industrial chemical production and food production. In the 19th century, people first observed microbes using microscopes and began to study them. However, the development of high-throughput sequencing technology has led to generation of large amounts of microbial related data. As a result, machine-learning methods are now being applied to microbiological research. Here, we discuss the current application of ML in the microbiome. The results revealed that ML is widely used in microbiological research, and that it has focused on classification problems and analysis of interaction problems. However, many problems remain unresolved and will require the cooperation of researchers from different fields, such as biology, informatics and medicine, to jointly promote the development and progress of microbiological research. On the other hand, the recent developed link prediction (Liu et al., 2016; Zeng et al., 2017b) and computational intelligence methods (Cabarle et al., 2017; Song et al., 2018), can be promising in discovering the relationship between diseases and microbes.

## Author Contributions

KQ drafted the manuscript. FG and XL conducted research. YL modified the manuscript. QZ conceived the idea.

## Conflict of Interest Statement

The authors declare that the research was conducted in the absence of any commercial or financial relationships that could be construed as a potential conflict of interest.
